# Subgrain Coalescence Simulation by Means of an Advanced Statistical Model of Inelastic Deformation

**DOI:** 10.3390/ma15155406

**Published:** 2022-08-05

**Authors:** Nikita Kondratev, Peter Trusov, Andrej Podsedertsev, Matvej Baldin

**Affiliations:** 1 Laboratory of Multilevel Structural and Functional Materials Modeling, Perm National Research Polytechnic University, 614990 Perm, Russia; 2 Mathematical Modeling of Systems and Processes, Perm National Research Polytechnic University, 614990 Perm, Russia

**Keywords:** high temperature deformation, subgrain structure evolution, subgrain coalescence, subgrain rotations, multilevel models

## Abstract

The development of technological methods for processing and manufacturing of functional (with a priori targeted properties) polycrystalline materials and products made of these materials still remains an acute problem. A multilevel modeling approach offers researchers the opportunity to describe inelastic deformation by applying internal variables that give an effective characterization of the material structure at different structural scale levels. High temperature plastic deformation is accompanied by these processes, which leads to a significant rearrangement of the meso- and microstructure of the material. The most substantial contribution to changing the properties of polycrystals is made by the evolution of grain and defect structures at the expense of dynamic recrystallization, which significantly depends on dynamic recovery. In this paper, we consider the problem of the coalescence of subgrains undergoing rotation during inelastic hot deformation. This process is called subgrain coalescence, and it is one of the dynamic recovery mechanisms responsible for changes in the fine subgrain structure. Under applied thermomechanical loads, the coalescence process promotes the formation of recrystallization nuclei and their subsequent growth, which can greatly change the grain structure of a polycrystal. The problem was solved in terms of the advanced statistical model of inelastic deformation, modified to describe the subgrain coalescence process. The model takes into account the local interactions between contacting structural elements (subgrains). These have to be considered so that the grain coalescence caused by a decrease in subboundary energies during their progressive merging can be adequately analyzed. For this purpose, a subgrain structure quite similar to the real structure was modeled using Laguerre polyhedra. Subgrain rotations were investigated using the developed model, which relies on the consideration of the excess density edge component of the same sign dislocations on incidental subgrain boundaries. The results of modeling of a copper polycrystal are presented, and the effects of temperature and strain rate on the subgrain coalescence process is demonstrated.

## 1. Introduction

Thermomechanical processing of metals and alloys has attracted growing interest because it has the potential to obtain ingots and final products with the required properties [1,2]. Experimental and theoretical studies provide numerous examples that indicate that the physical and mechanical properties of polycrystals (along with the operational characteristics of objects made of these materials) are determined by the state of the internal structure of the material [1,3,4,5,6]. Severe plastic deformation leads to significant rearrangements of the material structure at different scale levels, which causes the macroproperties of the material to change [1,5,7,8,9]. Most of the metal forming processes, especially in the manufacture of products from hard-to-form alloys, are realized at elevated and high temperatures [5,10]. Therefore, the complex structure change is determined predominantly by dynamic recrystallization (DRX) and dynamic recovery (DRV) [5,11,12,13,14]. Recrystallization includes the formation of new grains in the deformed material and the subsequent migration of high angle boundaries, the main driving force of which is the energy stored on the defects formed during inelastic deformation [15]. There are three main types of dynamic recrystallization: continuous [16,17], discontinuous [18,19] and geometric recrystallizations [20,21]. An important material parameter that determines the sequence of changes in the defect and subgrain structure is the stacking fault energy (SFE) ([5,12,13] etc.). As is known, the rearrangement of the dislocation structure in materials with a high SFE is accompanied by intense recovery, which is understood as a set of thermally activated processes. During these processes, the density of crystal defects decreases and subgrain structures with reduced energy are formed [5,11,12,13,22]. The intense recovery results in the formation of subgrain, cell-block and cell structures [5,12,22,23]. The processes of formation and rearrangement of the subgrain structure (polygonization, coalescence, migration and subgrain rotation) are determined mainly by the possibility of dislocation cross-slip. For intense cross-slip, along with elevated temperature, the increased SFE values are required [5]. This is followed by the homogeneous formation of nuclei, namely, their evolutionary transition to new grains, known as continuous recrystallization [16,17]. At low SFE values, the formation of recrystallization nuclei occurs in a heterogeneous way in the crystal lattice sites with significant distortions, and is called discontinuous or classical recrystallization [5,18,19]. Another type of recrystallization is geometric recrystallization which is associated with the material processing. In geometric recrystallization, the nucleation of elongated and thin (several subgrains thick) grains occurs, numerous “flaws” appear along the grain facets and the high angle boundaries are initiated, which finally results in the transition of grain parts to equiaxed new grains [13,20,21].

The main driving force of dynamic recrystallization and recovery is the energy stored on defects, therefore these processes are the competing ones [5,12]. During the thermomechanical treatment procedure, the recover process requires less activation energy than the recrystallization process [5,12]. For this reason, in high SFE materials, the recover process precedes recrystallization, thus preparing a subgrain structure for recrystallization. Recrystallization is a more efficient channel for releasing the energy stored on defects, therefore intense inelastic deformations and elevated temperatures ultimately lead to its activation. In some cases, especially in high SFE materials, the competition of these processes is more pronounced; continuous recrystallization is partially replaced by dynamic recovery [5,22,24]. The effect of recovery on the process of recrystallization is also observed in low and medium SFE materials. For example, due to the recovery effect, subgrains in copper polycrystals grow and the subgrain misorientation with the surrounding crystalline material increases [25,26,27]. Subgrain growth can occur in two different ways: by subgrain coalescence and by migration of subboundaries [5,24,28,29,30]. In this paper, we consider the coalescence process by which the adjacent subgrains merge together, due to the gradual disappearance of a common subgrain boundary during subgrain rotation [28,29,30]. In this way, the potential recrystallization nuclei may form and then transform into recrystallized grains [5,13,15,31]. The subgrain size is a dominating factor for recrystallization, since larger nuclei have the growth advantage and, accordingly, determine the subsequent grain structure evolution [5,18,32]. Despite extensive research in the field of recrystallization modeling [18,33,34,35,36], there is no common view and theory about the formation and growth of recrystallization nuclei. Thus, the recovery processes that affect the nucleation of recrystallized grains deserve theoretical studies that should involve mathematical modeling techniques. This paper investigates the influence of coalescence on the changes in the subgrain structure (misorientations and volumes of subgrains).

Calescence-related rotations occur due to the local interactions of the subgrain with the surrounding crystalline material. In this connection, the use of self-consistent models, in which the subgrain environment is replaced by an effective medium with averaged properties (see, for example, [37,38]) turns out to be impossible. For the first time, analysis of several flat sections of subgrain facets was made in [29,30,39]. Modeling of a representative volume of subgrains, with allowance for their geometry (that is close to the real three-dimensional geometry) is a complex and laborious task [5,40]. The central problem is related to the fact that the rotation of one subgrain changes the states of adjacent subgrains. These states affect the implementation of subsequent subgrain coalescence. An effective and flexible tool for studying the evolution of the material structure, in particular, the fine subgrain structure, is a multilevel modeling approach that involves the introduction of internal variables [1]. Even though the coalescence process has been known for a long time and has been confirmed by many experimental studies [28,29,30,41,42,43,44], only a few investigations of this process have been undertaken with physically based models of inelastic deformation designed to take into account the structure evolution of the material. The motivation of the present study is to develop a mathematical model capable of analyzing the evolution of misorientation and coalescence of subgrains, in terms of the advanced multilevel statistical model of high temperature inelastic deformation that has previously been developed [1,45,46]. The model has been modified to include the local interactions of the structural elements of the model (subgrains) with the surrounding elements.

## 2. Mechanisms Governing the Formation and Evolution of Subgrain Structure during Deformation

Plastic deformation that is mainly realized in polycrystalline materials at the expense of dislocation motion provides the hardening of these materials; the dislocation density also increases [7,47,48]. The uniform distribution of dislocations in the grain volume is not a steady-state and low-energy configuration. This results in the formation of dislocation structures in many materials subjected to plastic deformation. An example is the disordered dislocation substructures, whose configurations in the form of cell boundaries become clearer as the deformation proceeds [5,23,36]. Thus, the energy stored on dislocations tends to decrease due to the self-organization of dislocation arrays into low-energy structures [47,49]. The formation of low-energy structures most clearly manifested itself in the materials with high and medium SFE, which is determined by the occurrence of a recovery at different temperatures, including room temperature. The cell boundaries are saturated with dislocations, and therefore they are dislocation boundaries. The interiors of the cells are practically free of dislocation defects [5,23,50,51]. In crystals, dislocation structures are usually encountered in the form of two-dimensional dislocation boundaries, dislocation tangles, and three-dimensional dislocation cell structures [5,12,22,23]. In these configurations, as the cold deformation develops the dislocation density increases, and therefore the interaction energy of dislocations and the misorientation between parts of the crystal increase as well [5,47,52].

**Figure 1:**
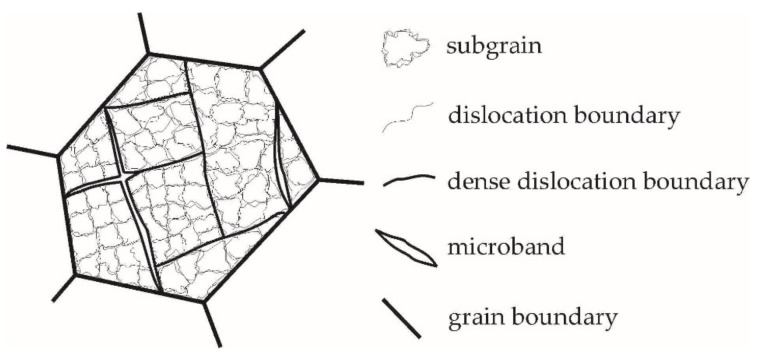
Scheme of the subgrain structure plotted according to the drawing from [23].

For many polycrystalline materials, the sequence of formation of dislocation configurations takes place at different scale levels [8,9]. According to the classification developed by D. Kuhlmann-Wilsdorf [53], the dislocation structure rearrangement during plastic deformation occurs at three scale levels. The smallest scale misoriented elements in a grain are the individual cells with a characteristic size of the order of a micrometer, which are separated by incidental dislocation boundaries or dislocation cell boundaries (Figure 1). A higher scale level represents blocks of cells that are the volumes of up to tens of adjacent cells with close orientation. The blocks are separated by dense dislocation boundaries or microbands (Figure 1). Microbands are the lamellar (long and thin) oriented regions which are limited by the grain sizes. The microband width is usually several tenths of a micrometer, and the microband length is from tenths to several tens of micrometers. The boundaries of cell blocks are more clearly defined on microsections, and the misorientation of blocks exceeds the misorientation of neighboring cells. Physically, the large misorientation of cell blocks, in comparison with neighboring cells, is attributed to the fact that the number of active slip systems in neighboring blocks is different. The microband boundaries consist of dislocation substructures similar to the cell boundaries but with a greater number of dislocations. As the deformation increases, the number of microbands and dislocation dense boundaries separating the initial homogeneous polycrystal grain increases. During plastic deformation, in contrast to the cell dimensions that remain almost unchanged, the cell block dimensions rapidly decrease, approaching one cell in the limiting case. Further deformation leads to the appearance of domains at a higher scale level. The number of these domains varies in the grain from 10 to 100 [53]. The domains are separated from each other by corresponding boundaries, which are called domain boundaries. Domains are the most misoriented scale elements, the dimensions of which significantly exceed the dimensions of cell blocks. In the neighboring domains as well as in the cell blocks, there are various active slip systems, the number of which usually varies from three to five.

For three scale levels of the subgrain structure, the following three types of boundaries are distinguished: cell boundaries, cell block boundaries and domain boundaries. These boundaries are the dislocation and low angle boundaries. The boundaries of cell blocks and domains are geometrically necessary boundaries, that appear when the corresponding parts of the grain rotate due to the movement and interaction of various slip system dislocations. The geometrically necessary boundaries also include the high angle grain boundaries, where the evolution of lattice rotations of adjacent grains is determined primarily by the incompatibility of plastic deformations [7]. At the initial stage of plastic deformation, the boundaries of individual cells are significantly different from the above-mentioned boundaries. They are the incidental boundaries because they are formed when the edge mobile dislocations are slowed down by the obstacles of a different nature, including forest dislocations [53]. Under plastic deformation, the misorientation between adjacent cells increases so that different slip systems begin to operate in these cells. This led us to the conclusion that the cell boundaries evolve into the geometrically necessary boundaries and become indistinguishable from the boundaries of cell blocks and domains. Such boundaries were called subgrain boundaries [53], and the elements separated by these boundaries are subgrains. The subgrain structure is formed at high displacement gradients [36].

**Figure 2:**
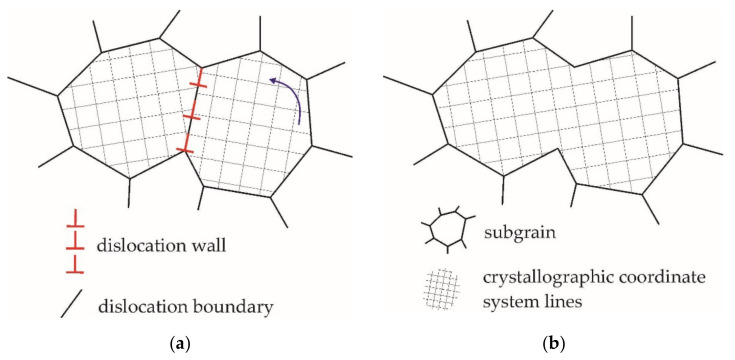
Scheme of subgrain rotation during coalescence: original subgrain structure (**a**), rotation of one of the grains (**b**) [29].

Temperature has a great effect on the subgrain structure evolution [5]. At elevated temperatures, the thickness of the cell boundaries, which in the limit consist of a single layer of dislocation networks [36], reduces and the dynamic recovery is activated. Subgrain growth can occur due to two processes: migration of low angle boundaries or coalescence [5,28,29,30]. The migration of a low angle boundary is induced by diffusion [30], as well as by the slip of dislocations located on the boundary [5]. Coalescence is realized through the rotation of adjacent subgrains when the low angle boundary between the subgrains disappears (Figure 2) [29,30]. During this process, the size of the subgrains and their misorientation with the surrounding crystalline material increases. Apparently, the subgrain coalescence mechanism was first discovered in the analysis of the experimental data on the annealing of Fe–Si alloy samples [28]. Previously, combined groups of subgrains were found inside the recrystallized aluminum grains [41] and subjected to annealing after the preliminary cold deformation, which is an indirect indication of coalescence. Further experimental observations also confirmed the presence of subgrain coalescence [42,43,44,54]. Although the in-situ experimental observations of coalescence have been carried out for a relatively long time, they remain laborious [55,56,57]. Thus, the assumptions about the subgrain growth mechanism (migration or coalescence) usually rely on indirect signs—the dislocation traces on microsections and the subgrain orientations observed via transmission electron microscopy or electron backscatter diffraction (EBSD) [5,58]. The results obtained in the microsection analysis are an acceptable experimental evidence for the subgrain coalescence process. The microsections under consideration show a large subgrain whose interior has two regions with a very small misorientation, the boundary of which is in the process of disappearing. The same subgrain structures have been determined by many researchers [28,41,42,43,44,58]. The main drawback of this method is that, during deformation, subgrains are in constant evolution, i.e., the old subgrains disappear and the new ones appear. The micrographs of the weakly developed low angle boundaries inside the clearly defined subgrains demonstrate with equal probability the disappearance of a subgrain boundary during coalescence and the formation of a new subboundary. By virtue of this, there is no uniform understanding of the subgrain coarsening mechanism, which can occur both due to coalescence and migration [5]. For example, in [59], an increase in the size of subgrains is associated with the migration of low angle boundaries. It was shown that, when the adjacent subgrains are misoriented by the angles in the order of 3–4°, the boundary migration proceeds much faster than the subgrain coalescence [15]. On the other hand, according to estimates made in [60], the influence of coalescence on the increase in the size of subgrains is great. Apparently, these two subgrain coarsening mechanisms are mutually reinforcing and complementary mechanisms which can proceed in parallel [30], since the characteristic migration and coalescence rates are close [30]. The subgrains coalescence is frequently observed near the high angle boundaries, deformation bands, and transition bands, which, at elevated deformation temperatures, promotes the formation of nuclei and the implementation of recrystallization [15,31,42,61].

The thermodynamic and kinetic aspects of the subgrain coalescence problem, which were formulated by J.C.M. Li [29], are still relevant today [5]. Based on the second law of thermodynamics, it was shown in [29] that the driving force of coalescence is a decrease in the surface energy of subgrains during their merging. Figure 2 presents a scheme of subgrain rotation by which the coalescence occurs. One of the subgrains rotates until the lattices coincide with the adjacent subgrain. Thus, a common low angle subgrain boundary is dissociated. As a result, the subgrains possess the same orientation and can be regarded as a single subgrain. It was shown [28,29,44] that coalescence can promote the evolution of subgrains into individual grains surrounded by a high angle boundary. In other words, the subgrain coalescence can be considered as a mechanism responsible for the formation of recrystallization nuclei. Due to ambiguity in interpreting the experimental data by a number of researchers, there is reason to doubt this conclusion [5]. However, the relevance of the study of coalescence has been confirmed by the results of many theoretical and experimental works in this field [42,43,44].

The subgrain rotation shown in Figure 2 leads to a change in the surface energy of all flat sections of its boundary, not only its common boundary with the adjacent subgrain. Thus, due to coalescence, the surface energy $E_{sb}$ of the subgrain undergoing rotation decreases [29]:

(1) $$E_{sb}=\sum_{i=1}^{N_{f}}s_{sb}^{\left(i\right)}e_{sb}^{\left(i\right)},$$

where $s_{sb}^{\left(i\right)}$ is the area of the *i*-th facet, $e_{sb}^{\left(i\right)}$ is the specific (per unit area) surface energy of the *i*-th facet, $N_{f}$ is the number of facets of the considered subgrain. For the small angle boundary, the specific surface energy is well described by the Read-Shockley relation [52]:

(2) $$e_{sb}=e_{sb0}\varphi \left(a-\ln\varphi \right),$$

where the following notation is used: φ is the angle of mutual misorientation between adjacent subgrains, and $e_{sb0}$, a are the experimentally obtained parameters of the Read-Shockley model.

An analysis that involves relation (1) was performed [29]. The obtained results demonstrate that: (1) the tilt boundaries, rather than the twist ones, predominantly occur; (2) due to the subgrain rotation, the size of facets with a relatively large area reduces; (3) the subgrain rotation promotes the elimination of the boundaries with a small misorientation angle and the increase in the share of higher misorientation, which in turn causes the large angle boundaries to appear. Various subgrain coalescence mechanisms are discussed in literature [5,29,30]: (1) the mechanism associated with the cooperative climb of dislocations into subboundaries. The boundary dissociation occurs due to an increase in the distance between dislocations on the boundary [29]; (2) the mechanism occurred due to the cooperative diffusion of atoms in the subgrain [29]; (3) the mechanism related to the extraction of dislocations from the subgrain boundary and their further annihilation, or the emission of dislocations, from the subgrain boundary [30]. During these processes, a common boundary between adjacent subgrains disappears and the role of a climb dislocation motion is significant in this case [30,39]. As reported in [39], the dislocations in the subgrain boundary are interconnected by loops, and therefore coalescence is possible only under the cooperative dislocation motion.

For the small angle boundaries with low misorientation, the mechanism of dislocation climbing and further annihilation [5,39,58] in a subgrain boundary seems to be more reasonable. An estimation expression is given [29] for determining the angular velocity of subgrain rotation, caused by coalescence under cooperative dislocation climbing (the crystallite index is omitted):

(3) $$\frac{d\varphi }{dt}=\left(3e_{sb0}\varphi Bb/s\right)\ln\left(\frac{\varphi }{\varphi_{m}}\right),$$

where B is the climb mobility parameter that has been obtained in the analysis of the kinetics of one isolated dislocation, with the maximum density of steps on it. In this case it can be taken equal to $Db/\left(2kT\right)$, D is the self-diffusion coefficient, k is the Boltzmann constant, T is the absolute temperature, and $\varphi_{m}$ is the mutual misorientation angle between adjacent subgrains. This corresponds to the maximum surface energy value in (2). Self-diffusion coefficients are determined through the Arrhenius-type relation [39,42]:

(4) $$D=D_{0}\exp\left(-\frac{Q}{RT}\right),$$

where $D_{0}$ is the pre-exponential factor of self-diffusion coefficient, Q is the activation energy of the self-diffusion process, and R is the universal gas constant. Experimental studies [42] revealed that during coalescence, the subgrain rotation rates are higher than those determined from relation (3). This discrepancy arises due to several reasons. First, during the dissociation of dislocation loops, the rotation rate for subgrain coalescence is primarily controlled by pipe diffusion [62] rather than by bulk self-diffusion [25,39]. Second, the uniform distribution of dislocations in the boundary, adopted by Li [29], gives a 4–10-fold underestimated value of the rotation rate [39]. The relation of dislocation loop climb mobility $B_{p}$ versus the pipe diffusion can be written as:

(5) $$B_{p}=\frac{2b^{3}D_{p}}{skT},$$

where $D_{p}$ is the pipe diffusion coefficient, which is defined by (4), the pipe diffusion activation energy $Q_{p}$ is 43% of the bulk self-diffusion Q [39]. Thus, according to (3), the amount of time that it takes to rotate during coalescence from the mutual misorientation angle $\varphi_{c}$ to the angle $\varphi_{0}$ is found as:

(6) $$t_{c}=\frac{s}{21e_{sb0}B_{p}b}\ln\left(\ln\left(\frac{\varphi_{m}}{\varphi_{0}}\right)/\ln\left(\frac{\varphi_{m}}{\varphi_{c}}\right)\right).$$

During the integration process similar to [29], the argument of logarithmic function was replaced by $\varphi_{m}/\varphi$. Relation (6) took into account the effect of reduction time $t_{c}$ at the expense of the inhomogeneous dislocation distribution on the subboundary, and the coefficient of 7 was added to denominator (6) [39]. The angle $\varphi_{0}$ in (6) was assumed to be equal to 0.000069 radians [39].

The description of the coalescence process provided above indicates a need to take into account the local interactions of a subgrain with its surrounding crystalline material, in order to create a physical model of coalescence. To this end, it is reasonable to consider both the topology of the distribution of crystallites in space and the change in the model inelastic deformation variables, which affect coalescence. The main physical quantity that determines the possibility of coalescence is the surface energy of subboundaries [29], which, according to the Reed-Shockley relation, is determined by the angle of mutual disorientation between adjacent subgrains [52]. Thus, a proper solution to the problem under study requires a mathematical model to investigate the contacting flat sections of the subgrain boundaries, their change during coalescence, as well as the rotation of subgrains during inelastic deformation. The current state of computer technology makes it possible to create physically based models of this type. In the next section, we describe the model developed for solving the problem that was just formulated.

## 3. Description of the Subgrain Orientation Evolution Using the Multilevel Statistical Model

Modeling of the subgrain structure evolution during plastic deformation is not a trivial task. Although there are vast amounts of theoretical data on this problem ([5,7,9,12,14,47] etc.), the whole variety of elements of this structure, as well as their evolution, cannot be fully described at the proper physical level [17,36,63,64]. In our study, a grain is represented by a set of homogeneous elements—crystallites. In the initial state of the material, a crystallite is associated with individual cells (Figure 1), which are separated by the incidental dislocation boundaries during the first stage of plastic deformation. For simplicity, these elements are hereinafter referred to as subgrains.

We use the advanced two-level model of inelastic deformation designed to take into account geometric nonlinearity [65]. A comprehensive description of the model is given [45,46]. The details of the mathematical formulations of physical models of inelastic deformation is provided in monograph [1]. For the purpose of the present investigation, we developed a variant of the two-level model [45,46]. This was modified to include the mechanism of subgrain coalescence during high-temperature inelastic deformation. In solving the formulated problem, we distinguish three structural-scale levels. They are macrolevel, mesolevel-I and mesolevel-II in the structure of the multilevel statistical model [45]. The macrolevel refers to a representative volume of the polycrystal containing a statistically significant number of grains. At mesolevel-I, a separate grain consisting of mesolevel-II elements (homogeneous subgrains) is considered. The grains are separated by the high angle boundaries, whose misorientation is more than 10–15°. At the same time, the subgrain misorientation varies from tens of minutes to several degrees [5,66].

**Figure 3:**
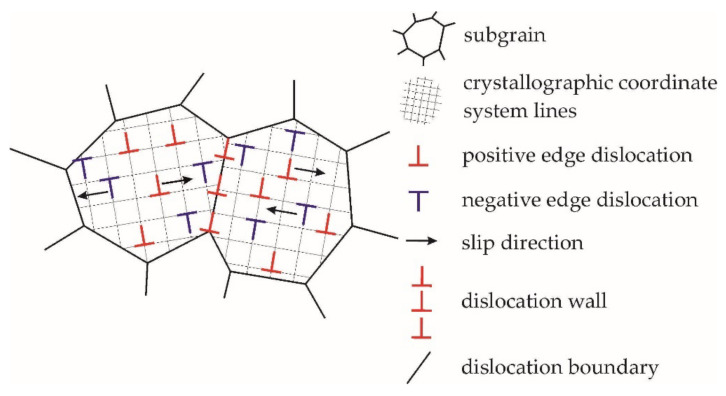
Scheme of the homogeneous plastic deformation and motion of edge dislocations of opposite signs along one slip system.

The relations of the mesolevel model are supplemented by the submodel equations describing subgrain misorientations. In [67,68], the authors proposed a method to analyze changes in the misorientation distribution of subgrains caused by to the formation and evolution of incidental low angle dislocation boundaries. The method suggests the use of physical multilevel models of inelastic deformation of a statistical type. For inelastic deformation, dislocations are assumed to be “trapped” on the initial incidental boundaries. Dislocations of opposite signs that interact on the boundary facet annihilate, and an excess density of dislocations of the same sign at the subgrain boundary leads to a rotation of one part of the crystal relative to the other (Figure 3). Defects such as dislocation dipoles were dropped from the consideration because they do not promote the rotations of crystal parts. It was also assumed that a larger fraction of edge dislocations β is retained on each flat section of the boundary from the side of the subgrain, and a smaller part of dislocations 1–β, which implement plastic deformation, is kept in the subgrain volume. The parameter β describes the state of the microstructure of individual subgrains, including their interior and boundary. At the initial stages of plastic deformation, the excess density of dislocations of the same sign on a common facet is determined by the difference in the values of the parameter Δβ of the contacting subgrains. The value of Δβ can take both positive and negative values, and the sign of Δβ determines the excess density of dislocations of a corresponding sign.

**Figure 4:**
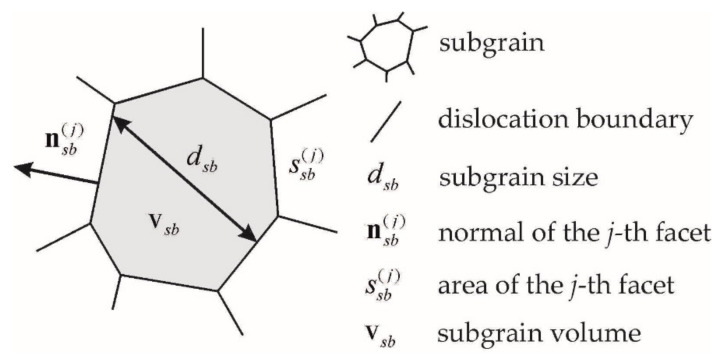
Scheme of the subgrain structure in the advanced statistical model.

A free software package, Neper, that is based on the consideration of Laguerre polyhedra is used to set initial subgrain boundaries [69]. A method for the formation of a grain structure and its transfer to the computational module of the statistical model (similar to that for subgrains) is presented in [70]. In the advanced statistical model, the obtained subgrain structure is determined by internal variables: the subgrain volume $v_{sb}$, the characteristic size $d_{sb}$ (determined by the diameter of the sphere of equivalent volume $v_{sb}$), the parameters of the flat sections of the boundary: the normal $n_{sb}^{\left(j\right)}$ and area $s_{sb}^{\left(j\right)}$, as well as an indication of neighboring grains to the considered grain (Figure 4).

For the small angle boundaries, the subgrain lattice misorientation angle $\varphi_{sb}$ is described with an appropriate degree of accuracy by the Burgers relationship [53,71]:

(7) $$\varphi_{sb}=b/d,$$

where b is the Burgers vector dislocation modulus, and d is the distance between the dislocations in the wall. The method of determining d via the internal variables of the two-level statistical model was developed [67]. To describe the subgrain rotation, we identified the edge dislocation component on the *j*-th facet of the boundary having the normal $n_{sb}^{\left(j\right)}$ with the excess density of dislocations of the same sign. The angular velocity vector $w_{d}^{\left(i,j\right)}$ of subgrain rotation caused by the accumulation of the *i*-th slip system dislocations on the considered facet $n_{sb}^{\left(j\right)}$ is determined by the relation [68]:

(8) $$w_{d}^{\left(i,j\right)}={\;\dot{\varphi }}_{d}^{\left(i,j\right)}l_{d}^{\left(i,j\right)}=\frac{1}{2}\Delta {\beta \;\dot{\gamma }}^{\left(i\right)}\cos\left(\alpha^{\left(i,j\right)}\right)l_{d}^{\left(i,j\right)},$$

where ${\;\dot{\gamma }}^{\left(i\right)}$ is the shear rate along the *i*-th slip system (determined using the physical elastoviscoplastic model), $l_{d}^{\left(i,j\right)}=n_{sb}^{\left(j\right)}\times n^{\left(i\right)}$ is the instantaneous axis of rotation, $n^{\left(i\right)}$ is the normal of the *i*-th slip system, $\alpha^{\left(i,j\right)}$ is the current angle between the considered normal of the *j*-th facet $n_{sb}^{\left(j\right)}$ and the Burgers vector $b^{\left(i\right)}$. The total rotation spin $\omega_{sg}$ is established by the summary rotation rate from all facets via trapping of dislocations over the active slip systems:

(9) $$\omega_{sb}=\frac{1}{s_{sb}}\sum_{j=1}^{N_{f}}\sum_{i=1}^{N_{a}}\omega_{d}^{\left(i,j\right)}s_{sb}^{\left(j\right)},\;\omega_{d}^{\left(i,j\right)}=-Є\cdot w_{d}^{\left(i,j\right)},$$

where the summation is over all active $N_{a}$ slip systems and over all facets $N_{f}$, Є is the Levi-Civita tensor, $s_{sb}^{\left(j\right)}$,$s_{sb}$ are the areas of the *j*-th facet and the area of the subgrain boundary, respectively, and $\omega_{d}^{\left(i,j\right)}$ is the rotation rate tensor caused by the accumulation of dislocations on the *i*-th slip system on the *j*-th facet. The total angular velocity vector $w_{sb}$ is determined by the total spin $\omega_{sb}$: $w_{sb}=\frac{1}{2}Є:\omega_{sb}$. The subgrain instantaneous axis of rotation $e_{sg}^{ins}$ coincides with direction of the vector $w_{sb}$: $e_{sb}^{ins}=w_{sb}/\sqrt{w_{sb}\cdot w_{sb}}$, the magnitude of the vector $w_{sb}$ determines the instantaneous rate of rotation ${\;\dot{\varphi }}_{sb}^{ins}=\sqrt{w_{sb}\cdot w_{sb}}$ about this axis.

In the framework of the rotation model just discussed, the influence of temperature on the subgrain rotation is considered indirectly, but it is explicitly taken into account by the relationships describing subgrain coalescence. In previous studies, it has been assumed that the dislocations of different signs on the boundary completely annihilate. The annihilation process is hindered by the fact that the dislocations in crystals are loops, and they accumulate on the boundaries in the form of tangles [36,47]. Thus, the annihilation requires the activation of a cross-slip of screw components of the dislocation loop. This process is thermally activated [5]. In the model of subgrain misorientation, the parameter β is responsible for the above effect. The study of the temperature dependence of this parameter is beyond the scope of this work. The description of determination of the parameter β is given in Section 4. The parameter β is calculated based on the experimental data of the subgrain orientation angles distribution at room temperature. If we have at our disposal the data obtained at different temperatures, then analysis of the dependence $\beta \left(T\right)$ is possible.

Under applied thermomechanical loads, the original homogeneous grain will experience different rotations of its subvolumes, i.e., it will undergo fragmentation accompanied by subgrain formation (Figure 1). To describe the subgrain orientation, we apply the misorientation tensor $r_{sb}$ which combines the orthonormal basis of the crystallographic coordinate system (CCS) of the subgrain $k_{sb}^{\left(p\right)}$ with the basis of CCS of the grain $k_{gr}^{\left(p\right)}$:

(10) $${\dot{r}}_{sb}\cdot r_{sb}^{T}=\omega_{sb},\;r_{sb}=\sum_{p=1}^{3}k_{gr}^{\left(p\right)}k_{sb}^{\left(p\right)},$$

where p is the number of the basis vector of the CCS of subgrain or grain; it varies from 1 to 3. The dyadic representation $r_{sb}$ given above is written for the basis of a Cartesian orthogonal coordinate system. At each instant of deformation, knowing the tensor $r_{sb}$, we can determine the misorientation angle $\varphi_{sb}$ and the axis $e_{sb}$ of the considered subgrain relative to the CCS of a grain:

(11) $$\varphi_{sb}=\arccos\left(\frac{I_{1}\left(r_{sb}\right)-1}{2}\right),\;e_{sb}=\frac{1}{2\sin\left(\varphi_{sb}\right)}Є:r_{sb},$$

where $I_{1}$ is the first principal invariant. To calculate the surface energy according to the Reed-Shockley relation (2), it is necessary to have the current angle of mutual misorientation between the adjacent *i*-th and *j*-th subgrains $\varphi^{\left(i,j\right)}$. To this end, we introduce the tensor $r_{m}^{\left(i,j\right)}$ of CCS misorientation between these subgrains:

(12) $$r_{m}^{\left(i,j\right)}={\left(r_{sb}^{\left(i\right)}\right)}^{T}\cdot r_{sb}^{\left(j\right)}.,$$

Using relations (11), the angle of mutual misorientation with the adjacent grain $\varphi^{\left(i,j\right)}$ is calculated for the tensor $r_{m}^{\left(i,j\right)}$. For each subgrain boundary, the surface energy $e_{sb}$ (along with the subgrain misorientation) are known from (2). The subgrain coalescence is modeled as follows. For the considered subgrain, the rotations determined by the tensor $r_{m}^{\left(i,j\right)}$ are calculated until the crystallographic lattices coincide with the adjacent subgrain. According to (1), the surface subgrain energy $E_{sb}\left(t\right)$ is calculated at the current instant of deformation *t* before rotation $r_{m}^{\left(i,j\right)}$ and after $E_{sb}^{r_{m}}$ imposed rotation (Figure 2). When the inequality is fulfilled:

(13) $$E_{sb}\left(t\right)\geq E_{sb}^{r_{m}}\left(t\right),$$

the surface energy diminishes and the coalescence process is feasible, i.e., the considered and adjacent subgrains can merge.

Coalescence proceeds via diffusion at the expense of the low angle boundary dissociation caused by the increased distance d between boundary dislocations. Under the assumption that the dislocations are in cooperative motion, the process time $t_{c}$ is determined by relation (6). In this case, in addition to energy criterion (13), the possibilities of boundary dissociation during the physical time $t_{c}$ should be assessed. To this end, the following procedure has been applied. The subgrain facet that satisfies (13) is associated with the known mutual misorientation angle $\varphi_{c}$ for the time $t_{c}$ required for the dissociation of this part of the subgrain boundary. If $t_{c}$ is shorter than the process time t, then it is assumed that the subgrain boundary facet can dissociate. If, at the current moment, several facets of the considered subgrain satisfy the coalescence implementation conditions, then the dissociation of the boundary which leads to the minimum value $E_{sb}^{r_{m}}\left(t\right)$ among all possible ones is accepted. The choice of a rotating subgrain is nontrivial; both subgrains can probably experience rotation under coalescence. After [29], we assume that only one subgrain undergoes rotation. We consider two possible options for the rotation of neighboring subgrains, and analyze the rotation of the subgrain which has the minimum magnitude of surface energy (13).

## 4. Model Identification, Simulation Results and Their Analysis

In this study, we investigate the inelastic deformation of a copper polycrystal in terms of an advanced statistical model for describing subgrain coalescence and rotation. The model parameters for these processes are given in Table 1.

**Figure 5:**
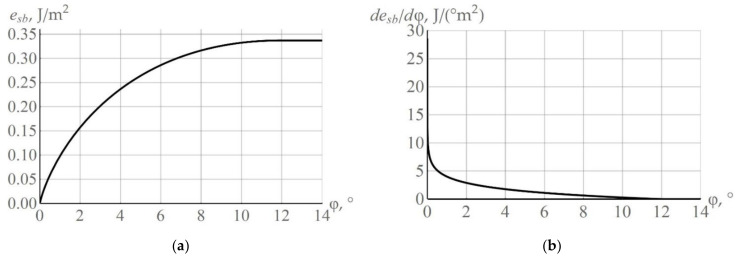
Curves illustrating the dependencies of the specific surface energy $e_{sb}\left(\varphi \right)$ (**a**) and $de_{sb}/d\varphi$ (**b**).

The surface energy between adjacent crystallites is determined by relation (2), which is applicable to small angle boundaries, i.e., up to the mutual disorientation angle φ in the order of 10–15° [52]. In this case, the dislocation cores do not overlap, and the correct relations are those of the linear theory of elasticity, which are used to obtain dependence (2). Most researchers suggest that, on reaching the angle $\varphi_{m}$, the intergranular energy is independent on the mutual misorientation angle φ, and it is a constant value ([40,75,76,77] etc.). We use here the same assumption: the angle $\varphi_{m}$ is determined under the condition $de_{sb}/d\varphi =0$, from which it follows that $\varphi_{m}=exp\left(a-1\right)$. For the considered material (copper) and the corresponding surface energy parameters (Table 1),$\varphi_{m}$ ≈ 11.92°, $e_{sb}\left(\varphi_{m}\right)$ ≈ 0.337 J/m^2^ are true. For copper, the dependences of the specific surface energy $e_{sb}\left(\varphi \right)$ and the derivative $de_{sb}/d\varphi$ are shown in Figure 5a,b, respectively. Analysis of the dependence $de_{sb}/d\varphi$ (Figure 5b) shows that, for the subgrains located near the high angle grain boundaries, a change in the surface energy of the high angle boundary facet is minimum in a coalescence rotation event. On the contrary, the largest changes in the surface energy magnitude $e_{sb}$ during rotations are expected for the subboundaries at small values of the angle φ. Therefore, the coalescence-driven subgrain rotations are the most probable events for the subgrains located near the grain boundary, and possess small misorientation angles with adjacent subgrains. Apparently, Doherty and Cahn were the first to turn their attention towards the problem of subgrain coalescence near high angle boundaries [15].

According to relations (3) and (6), the rotation rate dφ/dt and the rotation time $t_{c}$ during coalescence are determined by three main factors: (1) temperature T; (2) the angle of rotation during coalescence $\varphi_{c}$; and (3) the area of the common facet s. The values of $t_{c}$ and dφ/dt for the considered material (copper) at different T, $t_{c}$, s are summarized in Table 2. The data presented in the table indicate that the subgrains with small mutual misorientation angles merge together, mainly due to coalescence. The rotation time increases proportionally to the facet area, which is caused by the need to dissociate a larger number of dislocations on the given facet. Let us now analyze the following aspect of the considered mathematical model of coalescence. For the low angle boundary, during the arbitrary incidental rotation, there is a non-zero probability that the surface energy of the system (the entire representative volume of subgrains) will decrease. This can be verified via analyzing the Reed-Shockley dependence (2) or by conducting an appropriate numerical experiment. As the mutual misorientation angle φ increases, the surface energy, according to (2), increases more slowly compared to its reduction with a decreasing angle. When the dislocations are accumulated on the low angle boundaries during the formation of dislocation walls, or when their density decreases during coalescence, the rotations are the physically justified (not arbitrary) rotations. For the initial polyhedral structure of subgrains constructed in Neper, the number of facets is large and the average value is 13. This can be attributed to the high degree of sphericity (equiaxedness) of the subgrain structure. In this case, a subgrain has many facets, the area of which is a very small fraction against the total area of the boundary. The rotations along such facets are not justified as the physical phenomena involved in the coalescence process, and therefore they should be excluded from consideration, even though they lead to a decrease in the energy of the system (13). Test calculations provided evidence that the physically substantiated results for coalescence calculations can be obtained when the facet rotations, whose fraction is less than 15% of the total boundary area, were dropped from the study. Note that, according to the coalescence model, small subgrains, being attached to larger subgrains, form “clusters” and have more favorable rotation conditions.

**Figure 6:**
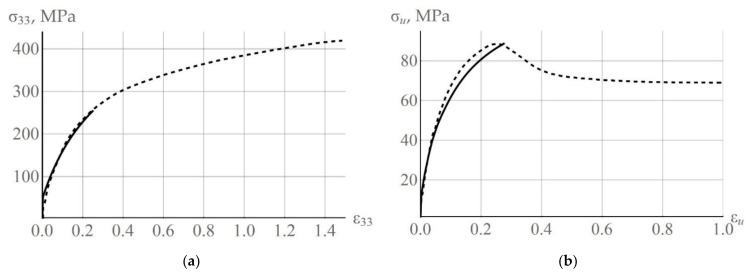
Loading diagram for the copper polycrystal at temperature 300 K and deformation velocity 10^−3^ s^−1^ (**a**) (dashed line—experiment [78]) at 775 K and velocity 2 × 10^−3^ s^−1^ (**b**) (dashed line—experiment [79]).

Hardening law parameters and initial critical stresses were determined based on the results of two uniaxial compression experiments on copper polycrystals, which were performed: (1) at a temperature of 300 K and deformation rate 10^−3^ s^−1^ [78]; (2) at a temperature of 775 K and deformation rate 2 × 10^−3^ s^−1^ [79]. For the high temperature experiment [79], the parameters of the hardening model were found prior to the active stage of recrystallization, i.e., at about 20% of deformation. Similarly, in this paper, we consider the computational experiments carried out at deformation values obtained before the onset of dynamic recrystallization, i.e., at 10% deformation. The experimental data used for parameter identification and the curves obtained with a multilevel model are shown in Figure 6. The initial critical stresses and hardening law parameters determined at different temperatures were approximated by a linear dependence. A comparison of the above results with the experimental data for the stress-strain state determined at room temperature [80,81,82] has revealed that in the considered range of strain rates 10^−5^–10^−3^ s^−1^, there is practically no dependence on this value.

In order to construct a subgrain structure in Neper, it is necessary to determine the statistical distribution laws for the sizes of subgrains $d_{sb}$ and their sphericity $\psi_{sb}$ (the ratio of the surface area of a sphere, whose volume is equal to that of the considered grain, to the surface area of the subgrain). The subgrain sizes in the reference configuration are assumed to be distributed according to the Rayleigh law with a 0.25 µm mean value [18,83]. The probability density of this law for the random variable takes the following form:

(14) $$f\left(x,\sigma \right)=\frac{x}{\sigma^{2}}\exp\left(-\frac{x^{2}}{2\sigma^{2}}\right),x\geq 0,$$

The parameter of the initial average value $d_{0sb}$ is determined from the parameter σ according to the well-known relation: $x_{0}=\sigma \sqrt{\pi /2}.$ The available experimental data demonstrate that the original subgrains have a shape that is close to an equiaxed shape [5,47], and therefore a uniform distribution of sphericity $\psi_{sb}$ with a high average value $\langle \psi_{sb}\rangle$ = 0.90 in the range from 0.85 to 0.95 was accepted. The application of Neper made it possible to create a polyhedral subgrain structure, whose geometric data were transferred to the calculation module of the statistical model.

**Figure 7:**
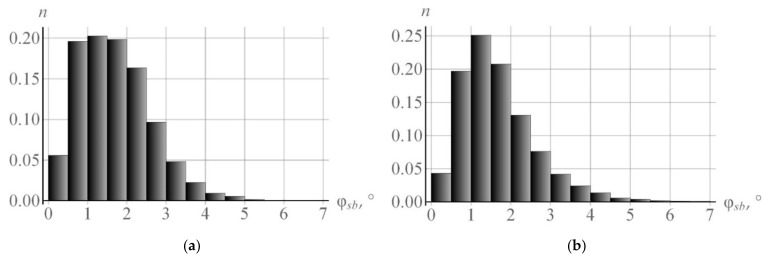
Distribution of the subgrain misorientation angles $\varphi_{sb}$ versus the grain CCS [66] found experimentally (**a**) and using the developed model (**b**).

The initial distribution of subgrain orientations and the submodel parameter Δβ for describing subgrain rotation were determined using the EBSD data [66]. The experimental histogram of distribution of the initial misorientation angles $\varphi_{sb}$ versus the grain CCS was approximated by the Rayleigh law (14). The average misorientation angle was $\varphi_{0}$ = 0.71°, and the maximum misorientation angle was 2.86° [66]. The rotation axis for setting the initial orientations of subgrains was generated uniformly at random on the sphere. To determine the parameter Δβ, the uniaxial tensile experiment on a copper polycrystal was performed at room temperature in which a copper sample was tensile deformed by 11% [66]. In view of the above reasoning, under the given temperature conditions, no coalescence occurs under the given temperature conditions. Thus, the parameter Δβ of subgrain rotation due to the accumulation of dislocations on random boundaries was determined independently of coalescence. Figure 7a gives an experimental histogram of the subgrain misorientation angles $\varphi_{sb}$ versus the grain CCS [66] at 11% deformation. Figure 7b presents the data in the angles $\varphi_{sb}$ obtained using the developed model.

The developed statistical two-level mathematical model of inelasticity is based on the Voigt hypothesis, where the prescribed kinematic effects are given by the velocity gradient $\hat{\nabla }V$:

(15) $$\hat{\nabla }V=\dot{\varepsilon }k_{01}k_{01}-\frac{\;\dot{\varepsilon }}{2}k_{02}k_{02}-\frac{\;\dot{\varepsilon }}{2}k_{03}k_{03},$$

where $k_{0i}=k_{0}^{i}$ is the orthonormal basis of the laboratory coordinate system, and $\;\dot{\varepsilon }$ is the prescribed deformation velocity. Temperature effects are transferred from the macrolevel, meaning the temperature T is a given temperature. Since the grain coalescence is the diffusion process, it is strongly affected by the temperature T. According to the proposed model, the deformation velocity $\;\dot{\varepsilon }$ also has a great influence on the coalescence process. At the high deformation velocity, the small angle boundary dissociation time $t_{c}$ can significantly exceed the deformation time. Further, we present the results of the computational experiments performed at deformation velocity $\;\dot{\varepsilon }$ from 10^−5^ s^−1^ to 10^−3^ s^−1^ and at a temperature from 350 to 750 K.

**Figure 8:**
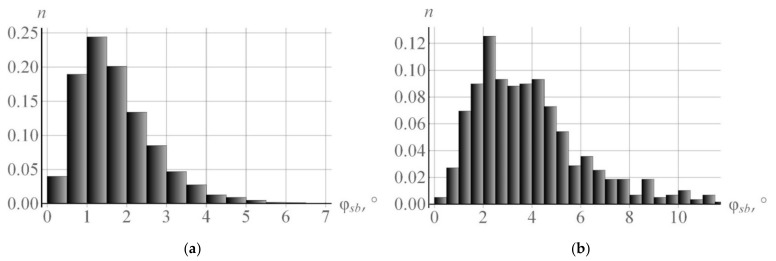
Histograms of distribution of the misorientation angles $\varphi_{sb}$ versus the grain CCS in the uniaxial tensile test at deformation velocity $\;\dot{\varepsilon }$ = 10^−4^ s^−1^ and temperature T = 350 K (**a**), at temperature T = 700 K (**b**).

Figure 8a gives a histogram of distribution of the misorientation angles $\varphi_{sb}$ versus the grain CCS during subgrain rotations (at the end of deformation). The histogram plot displays the accumulation of dislocations on the boundaries shown in the framework of the rotation model from Section 3 (deformation velocity $\;\dot{\varepsilon }$ = 10^−4^ s^−1^ and temperature T = 350 K). At this temperature, there is almost no subgrain coalescence: only 9950 out of the initial 10,000 subgrains remain at the end of deformation. The subgrain rotations are determined almost completely by the model of rotation due to the deposition of edge dislocations at low angle boundaries. In this case, the value of the mean angle $\langle \varphi \rangle$ was 1.76°. Figure 8b shows a histogram of angles $\varphi_{sb}$ for the same numerical experiment at temperature T = 700 K. In the second numerical experiment, the mean angle of mutual misorientation between subgrains $\langle \varphi \rangle$ increases due to intense coalescence and becomes equal to 3.93°. It is shown that the misorientation of subgrains increases, and the fracture of boundaries with small angles decreases due to coalescence. This corresponds to the conclusions [29].

**Figure 9:**
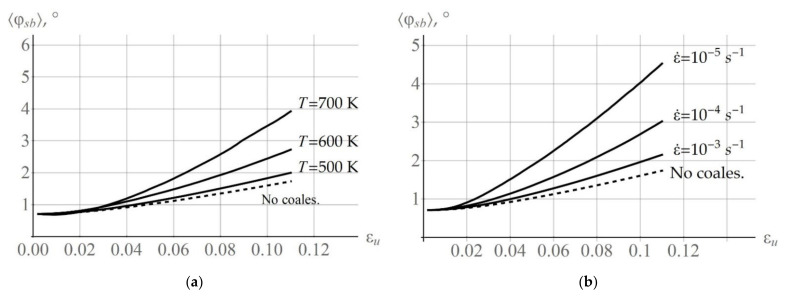
Evolution of the mean angle $\langle \varphi_{sb}\rangle$ in the uniaxial tensile test at deformation velocity $\;\dot{\varepsilon }$ = 10^−4^ s^−1^ and at different temperatures T = 500 K, 600 K, 700 K (**a**), at *T* = 550 K and different deformation velocities $\;\dot{\varepsilon }$ = 10^−5^, 10^−4^, 10^−3^ s^−1^ (**b**). Dashed line is evolution of the angle $\langle \varphi_{sb}\rangle$ without considering coalescence at temperature T = 500 K, $\;\dot{\varepsilon }$ = 10^−4^ s^−1^ (**a**) and T = 550 K, $\;\dot{\varepsilon }$ = 10^−3^ s^−1^ (**b**).

Figure 9a presents the curves showing the change in the mean subgrain misorientation angle $\langle \varphi_{sb}\rangle$, versus the CSC of grains caused by the accumulation of dislocations at low angle boundaries and due to coalescence at deformation velocities $\;\dot{\varepsilon }$ = 10^−4^ s^−1^ and at T = 500 K, 600 K, 700 K. Figure 9b illustrates the evolution of the angle $\langle \varphi_{sb}\rangle$ at the given temperature T = 550 K and at different deformation velocities: $\;\dot{\varepsilon }$ = 10^−5^, 10^−4^, 10^−3^ s^−1^. The obtained results indicate a significant contribution of coalescence to subgrain rotations at high temperatures and low deformation velocities. For comparison, Figure 9 presents the changes in the mean subgrain mutual misorientation angle $\langle \varphi_{sb}\rangle$ without considering coalescence at T = 500 K, $\;\dot{\varepsilon }$ = 10^−4^ s^−1^ (Figure 9a) and at T = 550 K, $\;\dot{\varepsilon }$ = 10^−3^ s^−1^ (Figure 9b).

**Figure 10:**
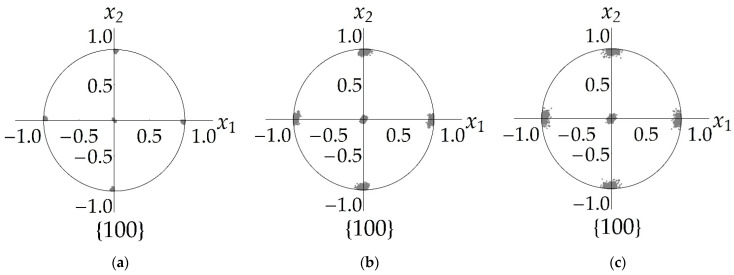
Direct pole figures {100} for reference configuration (**a**), the end of uniaxial deformation at velocity $\;\dot{\varepsilon }$ = 10^−4^ s^−1^ and at temperature T = 600 K without consideration of coalescence (**b**) and with consideration of coalescence (**c**).

Figure 10 presents the direct pole figures (DPF) of {100} planes which confirm the influence of coalescence on subgrain rotations. The DPFs {100} of the prescribed orientations of the subgrains CCS versus the grain CCS in the reference configuration are given in Figure 10a. The DPFs that were plotted using the data obtained at the end of uniaxial deformation at temperature T = 600 K and at $\;\dot{\varepsilon }$ = 10^−4^ s^−1^ without consideration of coalescence, are shown in Figure 10b and with consideration of coalescence in Figure 10c. It is seen that coalescence contributes to the increase in scatter of the CCS of subgrain orientations versus the grain CCS.

**Figure 11:**
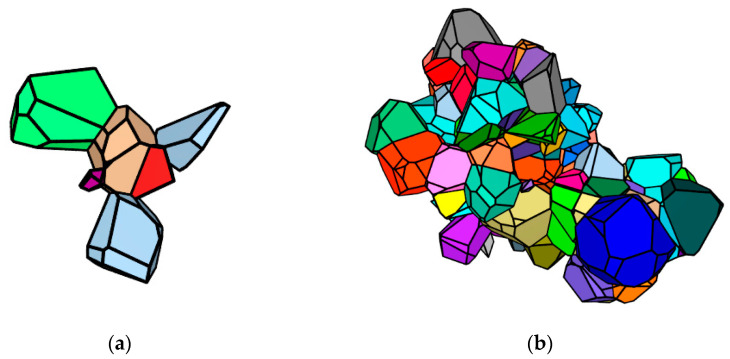
Subgrain clusters obtained in the numerical experiment on uniaxial deformation at $\;\dot{\varepsilon }$ = 10^−3^ s^−1^ and T = 550 K (**a**), at $\;\dot{\varepsilon }$ = 10^−5^ s^−1^ and T = 700 K (**b**).

During the coalescence event, subgrains form clusters (Figure 11). At deformation velocity $\;\dot{\varepsilon }$ = 10^−^^3^ s^−^^1^ and at the temperature T = 550 K, the calculated maximum cluster (Figure 11a) consists of 6 subgrains. For the event when $\;\dot{\varepsilon }$ = 10^−^^5^ s^−^^1^ and T = 700 K, the cluster includes 202 subgrains (Figure 11b).

**Figure 12:**
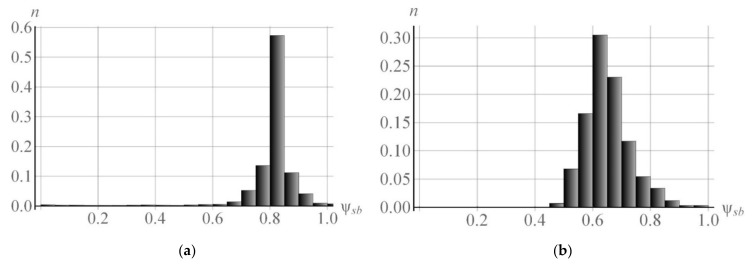
Histograms of distribution of subgrain sphericity $\psi_{sb}$ at the end of 11% deformation in the uniaxial tensile test at deformation velocity $\;\dot{\varepsilon }$ = 10^−4^ s^−1^ and temperature T = 550 K ($\langle \psi_{sb}\rangle$ ≈ 0.8) (**a**), $\;\dot{\varepsilon }$ = 10^−5^ s^−1^ and temperature T = 700 K ($\langle \psi_{sb}\rangle$ ≈ 0.64) (**b**).

Figure 12 presents the histograms of subgrain sphericity $\psi_{sb}$ at the end of deformation at T = 550 K, $\;\dot{\varepsilon }$ = 10^−4^ s^−1^ (Figure 12a) and at T = 700 K, $\;\dot{\varepsilon }$ = 10^−5^ s^−1^ (Figure 12b). Under these conditions, the mean sphericity values were $\langle \psi_{sb}\rangle$ ≈ 0.8 and $\langle \psi_{sb}\rangle$ ≈ 0.64, respectively. The resulting clusters of subgrains tend to decrease equiaxity with respect to the initial form. In a real crystal, the increase in equiaxity may be due to the migration of small angle boundaries, which was not taken into account in this work.

**Figure 13:**
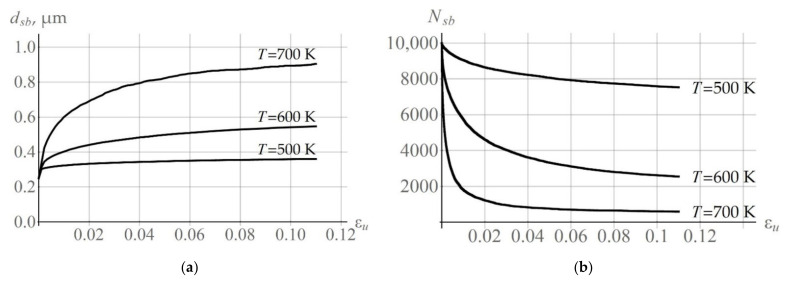
Evolution of the average subgrain size obtained in the numerical experiment uniaxial tensile test at deformation velocity $\;\dot{\varepsilon }$ = 10^−4^ s^−1^ and at different temperatures T = 500 K, 600 K, 700 K (**a**) and during variations in the number of subgrains (**b**).

The subgrain coalescence results in subgrain growth. Figure 13a shows how the average size $d_{sb}$ of the arbitrarily chosen grain increases at deformation velocity $\;\dot{\varepsilon }$ = 10^−4^ s^−1^ and at different temperatures T = 500 K, 600 K, 700 K. Figure 13b presents the calculated results illustrating the change in the number of subgrains in the arbitrarily chosen grain; the number decreases during coalescence. Simulations demonstrate that, at the initial stage of deformation, there are many subgrains that actively participate in coalescence. At the later deformation stage, the number of subgrain coalescence events reduce and the values of $d_{sb}$ and $N_{sb}$ tend to a steady-state value.

**Figure 14:**
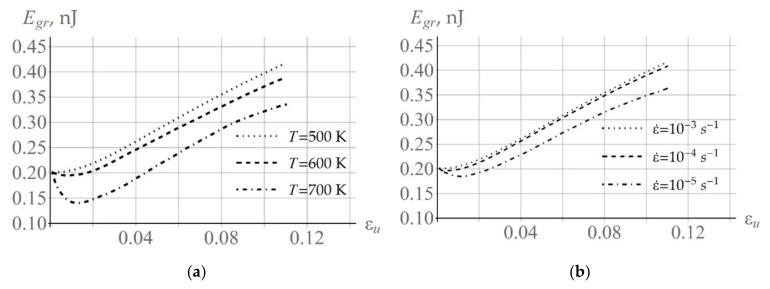
Subgrain boundary surface energy evolution for an arbitrarily chosen grain which was observed in the uniaxial tensile test: at $\;\dot{\varepsilon }$ = 10^−4^ s^−1^ and at T = 500 K, 600 K, 700 K (**a**); and at T = 550 K and deformation velocities $\;\dot{\varepsilon }$ = 10^−5^, 10^−4^, 10^−3^ s^−1^ (**b**).

According to (13), coalescence leads to a decrease in the total (over all subgrains) surface energy of the considered grain $E_{gr}$. The evolution of the surface energy of an arbitrarily selected grain $E_{gr}$ at various deformation velocities and temperatures is illustrated in Figure 14. It should be noted that, during plastic deformation, dislocations are deposited on the low angle boundaries, which enhances the misorientation between subgrains and increases the energy of the low angle boundary. Thus, the monotonic character of the dependence $E_{gr}$ depends on the processes described above. The drop in $E_{gr}$ at high temperatures and at low deformation velocities at the initial stage of plastic deformation is associated with intensive coalescence.

## 5. Discussion and Conclusions

In this paper, we propose a method for describing subgrain coalescence in terms of the advanced two-level statistical model. This model is designed to determine both the stress-strain state of the material, and the internal state variables characterizing the current state of the structure. The coalescence process leads to a change in the fine crystalline subgrain structure, where the local interaction effects play an important role. For this purpose, the initial polyhedral subgrain structure is formed in the software package Neper, and its image is transferred to the calculation module of the statistical model. The advantage of this model is that it takes into account the contact interactions of adjacent subgrains. The use of the developed rotation model makes it possible to calculate the subgrain rotations, and a specific subboundary energy corresponding to the mutual subgrain misorientation on each flat section of the subboundary. The low angle subboundary surface energy is the main physical factor (driving force) governing the coalescence process. The total surface energy of subgrains decreases due to coalescence. To calculate this value, information about the facet areas is required. These data (a subgrain geometry close to the real one, including the indication of adjacent subgrains and their states) are essential in calculating coalescence, and their absence makes the physical modeling of coalescence impossible.

During coalescence the subgrains form clusters, thus the average size of subgrains and their mutual misorientation increase. The simulations show that, at the initial instant of deformation, the subgrains (along with the low angle boundary facets with small areas) are subject to coalescence. The number of subgrains is sharply reduced under the appropriate temperature–velocity conditions of material processing. At further deformation stages, the dissociation of large area boundaries occurs, yet it develops with less intensity. Therefore, the effect of coalescence becomes noticeable at low deformation velocities or due to holding at an elevated temperature. The numerical experiments provided evidence that a decrease in sphericity is the result of intensive coalescence. This is primarily associated with the anisotropy of the considered medium—the surface energy of the subgrain facets is different. During a coalescence event, “competing” subgrain clusters occur that do not merge together during further deformation. These clusters have the reduced sphericity values compared to the initial value. Another aspect of this problem is related to the time constraints imposed on subgrain rotations, i.e., it takes more time to dissociate dislocations on the large area facets. Finally, we note that the model does not consider the migration of subgrain boundaries, which causes the subgrain sphericity to increase.

A separate problem, which is beyond the scope of this work and requires further investigation, is associated with the coalescence of subgrains near the high angle boundaries and transitional bands, where the misorientation with the surrounding material is large. For the high angle boundaries, the intergrain energy weakly depends on grain orientation (with the exception of special boundaries). Thus, due to coalescence, the subgrain rotations near the grain boundaries and transitional bands could have the advantage of merging. Under appropriate thermomechanical treatment conditions, the large subgrains (formed due to coalescence) with increased misorientations with the environment, are able to become the recrystallization nuclei. The calculated data obtained in this study indicate the importance of the coalescence process for the formation of a subgrain structure, and show its explicit dependence on temperature and deformation velocity. The modifications of the developed multilevel model can be used to study the recrystallization process by applying the coalescence-driven grain-growth mechanism described in this paper.

## Figures and Tables

**Table 1:** Copper parameters for describing the processes of coalescence and rotation of subgrains.

Parameter	Value	Literature Source
$e_{sb0}$	1.62 J/m^2^	[72]
a	–0.57	[72]
$\varphi_{m}$	0.208	Determined on the basis of the data from [72]
$\varphi_{0}$	0.000069	Determined on the basis of the data from [39]
$a_{lat}$	3.6 × 10^−10^ m	[73]
$b\approx 0.707a_{lat}$	2.55 × 10^−10^ m	Determined on the basis of the data from [73]
$D_{0}$	0.78 × 10^−4^ m/s^2^	[74]
Q	3.504 × 10^−19^ J	[74]
$Q_{p}=0.43Q$	1.507 × 10^−19^ J	Determined using the estimated value from [39] and the data from [74]
Δβ	±0.126	Determined on the basis of the data from [66]

In the table, the crystalline lattice period is denoted as $a_{lat}$

.

**Table 2:** Characteristic values of $t_{c}$ and dφ/dt for copper subgrain coalescence.

Parameters	T = 450 K, $\varphi_{c}$ = 1°, s = 0.1 µm^2^	T = 550 K, $\varphi_{c}$ = 1°, s = 0.1 µm^2^	T = 650 K, $\varphi_{c}$ = 1°, s = 0.1 µm^2^
$t_{c}$, dφ/dt	$t_{c}=112.38\times 10^{3}\;s$, $\frac{d\varphi }{dt}=1.46\times 10^{-6}\;s^{-1}$	$t_{c}=1.666\times 10^{3}\;s$, $\frac{d\varphi }{dt}=98.49\times 10^{-6}\;s^{-1}$	$t_{c}=0.09\times 10^{3}\;s$, $\frac{d\varphi }{dt}=1767.91\times 10^{-6}\;s^{-1}$
Parameters	T = 550 K, $\varphi_{c}$ = 2°, s = 0.1 µm^2^	T = 550 K, $\varphi_{c}$ = 3°, s = 0.1 µm^2^	T = 650 K, $\varphi_{c}$ = 4°, s = 0.1 µm^2^
$t_{c}$, dφ/dt	$t_{c}=2.13\times 10^{3}\;s$, $\frac{d\varphi }{dt}=196.99\times 10^{-6}\;s^{-1}$	$t_{c}=2.50\times 10^{3}\;s$, $\frac{d\varphi }{dt}=295.48\times 10^{-6}\;s^{-1}$	$t_{c}=2.83\times 10^{3}\;s$, $\frac{d\varphi }{dt}=393.97\times 10^{-6}\;s^{-1}$
Parameters	T = 650 K, $\varphi_{c}$ = 1°, s = 0.01 µm^2^	T = 650 K, $\varphi_{c}$=1°, s = 0.5 µm^2^	T = 650 K, $\varphi_{c}$ = 1°, s = 0.1 µm^2^
$t_{c}$, dφ/dt	$t_{c}=0.93\;s$, $\frac{d\varphi }{dt}=17679.11\times 10^{-6}\;s^{-1}$	$t_{c}=2.32\times 10^{3}\;s$, $\frac{d\varphi }{dt}=70.71\times 10^{-6}\;s^{-1}$	$t_{c}=9.28\times 10^{3}\;s$, $\frac{d\varphi }{dt}=17.68\times 10^{-6}\;s^{-1}$

## Nomenclature

The following abbreviations are used in this manuscript:

$\alpha^{\left(i,j\right)}$	angle between the normal of the *j*-th facet $n_{sb}^{\left(j\right)}$ and the *i*-th Burgers vector $b^{\left(i\right)}$
a	parameter of the Read-Shockley model
$a_{lat}$	crystalline lattice period
β	fraction of edge dislocations retained by the facet
Δβ	difference of fraction of edge dislocations retained by the facet
b	modulus of the Burgers vector
$b^{\left(i\right)}$	Burgers vector of the *i*-th slip system
B	climb mobility parameter
$B_{p}$	climb mobility parameter defined by pipe diffusion
d	distance between dislocations in the wall
$d_{sb}$	subgrain size
D	self-diffusion coefficient
$D_{p}$	pipe diffusion coefficient
$D_{0}$	pre-exponential factor of self-diffusion coefficient
$e_{sb}$	subgrain axis of rotation
$e_{sb}^{ins}$	subgrain instantaneous axis of rotation
$e_{sb}^{\left(i\right)},e_{sb}$	specific (per unit area) surface energy of the *i*-th facet
$e_{sb0}$	parameter of the Read-Shockley model
$E_{gr}$	surface energy of the grain
$E_{sb}$	surface energy of the subgrain
$E_{sb}^{r_{m}}$	surface energy of the subgrain after imposed rotation $r_{m}$
$\;\dot{\varepsilon }$	deformation velocity
Є	Levi-Civita tensor
$\varphi^{\left(i,j\right)},\varphi$	angle of mutual misorientation between the adjacent *i*-th and *j*-th subgrains
$\langle \varphi \rangle$	mean value angle of mutual misorientation subgrains
$\varphi_{0}$	minimum angle of mutual misorientation between adjacent subgrains
$\varphi_{c}$	current angle of mutual misorientation between adjacent subgrains
$\varphi_{m}$	mutual misorientation angle between adjacent subgrains, which corresponds to the maximum surface energy
${\;\dot{\varphi }}_{sb}^{ins}$	instantaneous rate of the subgrain rotation
$\varphi_{sb}$	misorientation angle between CCS of subgrain and grain
${\;\dot{\gamma }}^{\left(i\right)}$	shear rate along the *i*-th slip system
k	Boltzmann constant
$k_{gr}^{\left(p\right)}$	basis vector of the CCS of the grain
$k_{sb}^{\left(p\right)}$	basis vector of the CCS of the subgrain
$k_{0i},k_{0}^{i}$	vector of orthonormal basis of the laboratory coordinate system
$l_{d}^{\left(i,j\right)}$	instantaneous axis of rotation caused by the accumulation of the *i*-th slip system dislocations on the *j*-th facet
$n^{\left(i\right)}$	normal of the *i*-th slip system
$n_{sb}^{\left(i\right)}$	normal of the *i*-th facet
$N_{a}$	number of active slip system
$N_{f}$	number of facets of the subgrain
$\psi_{sb}$	sphericity of the subgrain
$\langle \psi_{sb}\rangle$	mean value sphericity of the subgrains
Q	activation energy of the self-diffusion process
$Q_{p}$	activation energy of the pipe-diffusion process
$r_{sb}$	misorientation tensor of CCS of the subgrain
$r_{m}^{\left(i,j\right)},r_{m}$	CCS misorientation between *i*-th and *j*-th subgrains
R	universal gas constant
$s_{sb}^{\left(i\right)},s$	area of the *i*-th facet
$s_{sb}$	area of the subgrain boundary
$t_{c}$	time of rotation during coalescence from the mutual misorientation angle $\varphi_{c}$ to the angle $\varphi_{0}$
T	absolute temperature
$v_{sb}$	subgrain volume
$w_{sg}$	total angular velocity vector of subgrain
$w_{d}^{\left(i,j\right)}$	angular velocity vector of subgrain rotation caused by the accumulation of the *i*-th slip system dislocations on the *j*-th facet
$\omega_{sg}$	total rotation spin of subgrain
$\omega_{d}^{\left(i,j\right)}$	rotation rate tensor caused by the accumulation of dislocations on the *i*-th slip system on the *j*-th facet
$\hat{\nabla }V$	velocity gradient
CCS	crystallographic coordinate system
DPF	direct pole figures
DRV	dynamic recovery
DRX	dynamic recrystallization
EBSD	electron backscatter diffraction
SFE	stacking fault energy
